# Health hazards of particles in additive manufacturing: a cross-disciplinary study on reactivity, toxicity and occupational exposure to two nickel-based alloys

**DOI:** 10.1038/s41598-023-47884-1

**Published:** 2023-11-27

**Authors:** Hanna L. Karlsson, N. V. Srikanth Vallabani, Xuying Wang, Maria Assenhöj, Stefan Ljunggren, Helen Karlsson, Inger Odnevall

**Affiliations:** 1 Institute of Environmental Medicine, Karolinska Institutet, 171 77 Stockholm, Sweden; 2 Occupational and Environmental Medicine Center in Linköping, and Department of Health, Medicine and Caring Sciences, Linköping University, Linköping, Sweden; 3 KTH Royal Institute of Technology, Division of Surface and Corrosion Science, 100 44 Stockholm, Sweden; 4 AIMES - Center for the Advancement of Integrated Medical and Engineering Sciences, Karolinska Institutet and KTH Royal Institute of Technology, Stockholm, Sweden; 5 Department of Neuroscience, Karolinska Institutet, 171 77 Stockholm, Sweden

**Keywords:** Chemical biology, Health occupations, Risk factors, Materials science

## Abstract

The increasing use of additive manufacturing (AM) techniques (e.g., 3D-printing) offers many advantages but at the same time presents some challenges. One concern is the possible exposure and health risk related to metal containing particles of different sizes. Using the nickel-based alloys Hastelloy X (HX) and Inconel 939 (IN939) as a case, the aim of this cross-disciplinary study was to increase the understanding on possible health hazards and exposure. This was done by performing in-depth characterization of virgin, reused and condensate powders, testing in vitro toxicity (cytotoxicity, genotoxicity, oxidative stress), and measuring occupational airborne exposure. The results showed limited metal release from both HX and IN939, and slightly different surface composition of reused compared to virgin powders. No or small effects on the cultured lung cells were observed when tested up to 100 µg/mL. Particle background levels in the printing facilities were generally low, but high transient peaks were observed in relation to sieving. Furthermore, during post processing with grinding, high levels of nanoparticles (> 100,000 particles/cm^3^) were noted. Urine metal levels in AM operators did not exceed biomonitoring action limits. Future studies should focus on understanding the toxicity of the nanoparticles formed during printing and post-processing.

## Introduction

The use of three‐dimensional (3D)-printing or “additive manufacturing” (AM) techniques to manufacture metallic (and polymeric) components is rapidly expanding and will involve an increasing number of workers in the near future. These technologies enable complex 3D components to be manufactured by adding layer upon layer of materials such as different metal powders. The use of AM technology offers many advantages, for instance by generating less waste, decreasing the overproduction of products, reducing the need for storage space due to possibilities for printing “on demand” and aiding start-up companies in developing countries due to the higher flexibility compared to traditional manufacturing. These AM technologies are viewed as highly important for achieving the sustainable use of critical raw materials^1–3^. At the same time, AM also presents unique potential challenges regarding occupational health because of a large variety of processes and the increasing use of new types of materials^4,5^. AM techniques that use metallic powders include binder jetting (a binding liquid is deposited to join the powder material), and powder bed fusion (PBF) techniques^6^. The PBF techniques use either a laser beam (PBF-LB) or electron beam (PBF-EB) to melt and fuse the powders. The specific AM-technique and type of printer show different risks for particle exposure to the workers, depending if they are, or not, equipped with a closed powder handling system. Indeed, particle exposure can occur during handling of powder feedstocks used for AM printers, but particles can also be generated during the AM process^7^. Thus, inhalation and dermal exposure can occur at different stages of the production, including not only powder handling but also printing, post-processing, machine cleaning, and maintenance^4,8^. It has been highlighted that more investigations are needed to understand which tasks contribute the most to workers’ exposure^5^.

There are several uncertainties that make it difficult to clearly understand the health risks for workers in AM. One of them concerns how to consider the effects of different particle sizes when measuring the exposure. Graff et al.^9^ showed, for example, that nano-sized particles were present in the AM environment and that operators were specifically exposed when handling the metal powder. Ljunggren et al.^10^ also reported that AM operators showed relatively low dust exposure when gravimetric analyses were used, but, at the same time, particle-counting instruments (analyzing particles from 10 nm to 10 µm) showed peaks with large numbers of nanosized particles generated during specific work tasks in the AM facilities, and upon post-processing. Thus, only the use of gravimetric measures may underestimate the risk of particle exposure. Exposure to particles can cause health concerns since several metallic powders used in AM contain metals such as chromium (Cr), nickel (Ni), and cobalt (Co), which might cause various health effects such as allergic contact dermatitis and lung cancer upon human exposure^11–13^. After lung deposition of particles, their health effects and fate are governed by the particle characteristics and surface composition including their solubility/metal release and toxicity. The released metals can cause toxicity locally in the lung but may also be absorbed, leading to effects at secondary sites^14^. Thus, some particles can dissolve after deposition in the lung, either in the lung lining fluid or in the acidic lysosomal fluid after cellular uptake, whereas other particles may remain in the particulate form and release metals at a much slower rate^15^. Such poorly soluble particles may result in chronic effects^16^.

Another uncertainty is the lack of understanding of the physical–chemical and toxicological characteristics of the particles used (virgin and reused) and generated (condensate and spatter) during the printing activities, including the extent to which the reuse of the powders affects their properties. Since not all powder particles are fused during the process, non-fused powders within the powder bed are reused, often several times, and mixed with virgin powders. Some studies on reused metal alloy powders have been performed and many of them show minor changes in terms of particle size distribution and bulk composition, but a slight increase in oxygen content compared with virgin powders, whereas other alloys show larger changes in physical–chemical properties and microstructure^8,17^. However, changes in the surface oxide composition and differences in bioaccessibility (metal release), as well as toxicological properties have been less explored. We recently investigated virgin and reused powders of three iron- and nickel-based alloy powders (316L, IN718, 18Ni300) in terms of metal release in artificial lysosomal fluid (ALF) and cytotoxicity (10–100 µg/mL)^16^. The results showed that less than 1% (< 0.01 µg/µg) of the particle mass was dissolved from the alloys forming passive surface oxides (316L, IN718), whereas the 18Ni300 iron-nickel alloy with a less corrosion resistant oxide dissolved completely within 24 h^17^. When comparing virgin and reused powders, the results showed minor differences in surface oxide composition and metal release for 18Ni300 and IN718, whereas the 316L powder showed an enrichment of manganese within the outermost surface and an increase of released iron. All powders showed low, or negligible, cytotoxic potency^17^. In another recent study, we focused on spatter/condensate particles and showed also low cytotoxic, genotoxic, and inflammatory potential of these particles^18^. On the other hand, subtle morphological changes, such as concentration-dependent effects on the cytoskeleton were observed in a recent study on iron-based AM particles^19^. More studies are needed regarding inhalable particles (< 4 µm) that more easily can reach deep into the lung.

Using powder particles of nickel-based Hastelloy X (HX) and Inconel 939 (IN939) alloys as a case, the aim of this cross-disciplinary study was to increase the understanding of their possible health hazards by studying occupational exposure and characteristics of the powders. More specifically, this was done by (1) performing an in-depth characterization of virgin, reused and condensate powders; (2) testing the in vitro toxicity with focus on “dusted” powders collected on filter as well as a condensate formed during printing; (3) measuring the occupational airborne exposure levels of particles of different sizes during various work tasks; and (4) assessing metal levels in urine in a selected number of workers exposed to HX and/or IN939.

## Methods

### Chemicals

All chemicals used in this study for metal analysis were of analytical grade, including citric acid, NaOH, NaCl, CaCl_2_·H_2_O, disodium tartrate dihydrate, sodium pyruvate, sodium citrate, trisodium citrate dihydrate, Na_2_HPO_4_, glycine, MgCl_2_, and Na_2_SO_4_. Ultrapure water (resistivity—18.2 MΩcm, Millipore, Solna, Sweden) was used as a solvent (for solution preparation) and cleaning agent (for acid-cleaning) throughout this study. For cell assays, the following chemicals from Sigma-Aldrich (St. Louis, MO) were used: 2′,7′-Dichlorofluorescin diacetate (DCFH-DA), NaCl, Tris, EDTA, Triton X-100, NaOH, SYBR Green and Tris–acetate-EDTA (TAE) buffer.

### Particles

Virgin (as received feedstock) and reused non-fused gas-atomized metal alloy powders of the nickel-based superalloy Hastelloy X and the nickel–chromium based alloy Inconel 939 (IN939) were investigated. The virgin alloy powders are commercially available (obtained from EOS store, Germany) and were provided by Siemens Energy. HX has a composition of 47 wt.% nickel (Ni), 22 wt.% chromium (Cr), 18 wt.% iron (Fe), 9 wt.% molybdenum (Mo), 1.5 wt.% cobalt (Co), and other metals (below 1 wt.%). IN939 has a composition of 50 wt.% Ni, 22.5 wt.% Cr, 19 wt.% Co, 3.7 wt.% titanium (Ti), 2 wt.% tungsten (W), 1.9 wt.% aluminum (Al), 1.4 wt.% tantalum (Ta) and 1 wt.% niobium (Nb). Mixtures of condensate/spatter particles following HX and IN939 printing were also included (denoted as “condensates”). These samples were collected after PBF-LB printing from the printing chamber from the walls of the chamber (except from the bottom) and from the outlet of the chamber. The HX condensates of different size fractions were sieved and kindly provided by Siemens Energy. To test particles with a smaller size-fraction, particles sized < 10 μm (Particulate Matter, PM_10_) of the reused powders, HX and IN939 particles were manually “dusted” (5 kg of reused HX and IN939 powder supplied from the AM site), by using a high-volume sampler (HVS; HiVol3000, Ecotech). The HVS was placed inside an exposure chamber and the powder was manually poured from one vessel to another using a sieve to enable substantial dusting. The dusted powder (> 100 mg) was collected on glass fibre filters (Pallflex PTFE, Melbourne, Australia). Sampling was conducted for 6 h at an air flow of 1.1 m^3^/min.

### Bioaccessibility studies

Bioaccessibility studies were conducted as previously described in Wang et al.^17^. In short, triplicates of the powders with one corresponding blank sample (without powder) were exposed to artificial lysosomal fluid (ALF, pH 4.5, see details in supporting information) at 37 °C for 24 h at a particle mass to solution volume ratio (loading) of 0.1 g/L. All exposures were conducted at dark conditions in an incubator using gentle bilinear agitation (inclination 12°, 22 cycles/min). After exposure, the upper part of the test solution was transferred to an acid-cleaned test tube and centrifuged (3000 g for 10 min) to separate the particles from the solution. The particle-free supernatant was then transferred to another acid-cleaned tube and acidified using 65% HNO_3_ to a pH less than 2 for metal release analysis. Total amounts of released Co, Mo, Cr, Ni, W and Fe in solution from HX were analyzed by means of inductively coupled plasma sector field mass spectrometry (ICP-SFMS; ELEMENT 2, Thermo Scientific,Luleå, Sweden). The determination limits of the metals of interest in the ALF solution were 0.05 µg/L for Co, 0.2 µg/L for Mo, 0.5 µg/L for Cr, and Ni, 0.05 µg/L for W and 10 µg/L for Fe. All reported data reflects mean values of three independent samples for each powder with the respective blank sample subtracted. Further experimental details are given elsewhere^17^. Released amounts of Ni and Co from IN939 were determined by means of graphite furnace atomic absorption spectroscopy (GFAAS; PerkinElmer PinAAcle900T, Stockholm, Sweden).

### Particle and surface characterization

The morphology, shape and size of powder particles were characterized by means of scanning electron microscopy (SEM; FEI XL30 SEM and INCA software, 20 kV, Stockholm, Sweden). The powders were fixed onto carbon tape to ensure their best electrical conduction. Surface oxide (5–10 nm) compositional analysis were performed using X-ray photoelectron spectroscopy (XPS, UltraDLD spectrometer, Kratos Analytical, Stockholm, Sweden) using a monochromatic Al Kα X-ray source (150 W). Overview and detailed high resolution XPS spectra (20 eV pass energy) were acquired for C 1s, O 1s, Cr 2p, Ni 2p, Co 2p, Fe 2p, Ti 2p, Nb 3d, V 4f, Ta 4f, W 4f and Mo 3d. The binding energies were calibrated versus adventitious carbon at 284.8 eV. The results are presented as the relative amount of oxidized metals in the outermost (7–10 nm) surface oxides. The corrosion resistance (i.e., barrier properties) of the surface oxide of the HX powders was investigated in ALF using a PARSTAT MC Multichannel Potentiostat (Princeton Applied Research, Stockholm, Sweden). Potentiodynamic polarization was conducted using an Ag/AgCl saturated KCl electrode as reference- and a platinum wire as counter electrode. More experimental details are given in Wang et al.^17^.

### Sample preparation and cell culture

As previously described^18^, the powders were weighed and dispersed in Milli-Q water to a final concentration of 1 mg/mL followed by sonication (Ultrasonic cleaner model USC 200T, frequency 45 kHz, VWR, Stockholm, Sweden) for 20 min at 30 °C using a water bath sonicator and then further diluted in cell medium (see below) to the indicated concentrations (10–100 µg/mL). In case of dusted HX and IN939, filter pieces of 2.25 cm^2^ (1.5 × 1.5 cm) were cut and weighed. Next, filters were placed on sides in a 5 mL glass vial (VWR). Gently, 1 mL of Milli-Q water was added drop by drop onto the filters to collect particles (carefully and quickly as they release fibers otherwise). After this, filters were removed, dried for 2 h and re-weighed. The concentrations of particles in the water suspension were based on the difference in filter weight before and after extraction. Similarly, blank filters were used to consider the background effect from fibers. The particle suspensions were sonicated for 20 min at 30 °C and then further diluted to the final desired concentration in cell culture medium. Fresh suspensions were prepared before each experiment. Human bronchial epithelial cells (HBEC3-kt, originally obtained from the American Type Culture Collection) were cultured in flasks pre-coated with 0.01% collagen (Type I, PureCol, Advanced BioMatrix, Carlsbad, California, USA) in 50% LHC-9 (Laboratory of Human Carcinogenesis-9, from Gibco, Fisher Scientific, Göteborg, Sweden) medium and 50% RPMI-1640 (Roswell Park Memorial Institute, Gibco, Sweden) medium. The medium was supplemented with 1% penicillin–streptomycin (PEST, Gibco, Sweden) and 1% l-glutamine but no serum was added. Cells were seeded at desired density as specified below 24 h prior to exposure on pre-coated plates. The cells were kept in a humidified atmosphere at 37 °C, 5% CO_2_ and sub-cultured at 80% confluency.

### Cell viability

Alamar blue assay was performed to determine the cytotoxic potential of the particles following exposure of HBEC cells for 24 and 48 h. Briefly, 1 × 10^4^ cells/well were seeded in precoated transparent 96 well plates (Corning 3598). After 24 h, the medium was removed and 100 µL of particles suspensions was added in triplicate wells in the concentrations 10, 25, 50, 75 and 100 µg/mL. NiO nanoparticles (Sigma-Aldrich, St. Louis, MO) at concentration 100 µg/mL was used as positive control. After exposure, the medium was removed from the wells and 100 µL of 10% Alamar blue reagent (Invitrogen, Carlsbad, CA) in cell medium was added to each well followed by incubation for 2 h at 37 °C. The fluorescence was then measured using a Tecan Infinite F200 plate reader (Tecan, Magellan 7.2 Software, Grodig, Austria) at 540/590 nm excitation and emission. Cell-free blank (Alamar blue with the particles) was included to enable subtraction of the background. The negative control (cells in medium, or in medium with blank filter extract, respectively) was set to 100% viability and the mean fluorescence of treated samples was normalized to this value. The results were expressed as % viability compared to control cells.

### Intracellular ROS

Intracellular ROS generation was assessed by using 2′,7′-Dichlorofluorescin diacetate (DCFH-DA, Sigma-Aldrich, St. Louis, MO). DCFH-DA is a lipophilic, non-fluorescent cell-permeable dye that gets deacetylated to DCFH in the presence of cellular esterases. DCFH will after oxidation by free radicals turn to highly fluorescent 2′,7′-dichlorofluorescein (DCF). The cells were seeded at 1 × 10^4^ cells/well in pre-coated black well plates (Costar, clear bottom). After 24 h, they were exposed to 10, 50 and 100 µg/mL particles for 3 h. Fe_3_O_4_ nanoparticles (50 µg/mL, synthesized as described by Vallabani et al.^20^) was used as positive control. Next, the cells were gently washed with PBS (phosphate buffered saline, Thermo Fisher Scientific, Waltham, MA) and incubated with 25 μM DCFH-DA for 45 min at 37 °C. Thereafter, the cells were washed with PBS and 100 µL of PBS was added to each well and fluorescence intensity was recorded using a plate reader (Tecan Infinite F200) at an excitation and emission of 485/535 nm respectively. The negative control (cells in medium, or in medium with blank filter extract, respectively) was set to 100% and the mean fluorescence of treated samples was normalized to this value. The results were expressed as % ROS compared to control cells.

### DNA strand breaks

The alkaline version of the comet assay detecting DNA strand breaks and alkali labile sites were used. Cells were seeded at 6 × 10^4^ cells/well in 24-well plates (Costar) and were then exposed to particles in the concentrations 10, 50 and 100 µg/mL for 3 and 24 h, respectively. H_2_O_2_ (100 μM for 5 min on ice) was used as a positive control and cells in medium, or in medium with blank filter extract, respectively, was used as negative control. After exposure, medium was removed, and cells were harvested and washed with PBS. Approximately 1 × 10^4^ cells were then embedded in 0.75% low melting agarose (LMP, Sigma-Aldrich, St. Louis, MO) that was spread over a microscopic slide precoated with 0.3% agarose. Next, the cells were lysed in lysis buffer (2.5 M NaCl, 10 mM Tris, 0.1 M EDTA, with pH 10) containing 1% Triton X-100 at 4 °C, overnight. Slides were then emerged in the electrophoretic buffer (0.3 M NaOH, 1 mM EDTA) to unwind DNA (for 40 min), and electrophoresis was performed for 30 min at 29 V. After electrophoresis, the cells were neutralized (0.4 M Tris for 5 min twice and in water for 5 min), dried overnight, and fixed in methanol for 5 min. Finally, the cells were stained with SYBR Green (1:10,000 dilution in TAE buffer) for 20 min, washed with TAE and dried completely prior to scoring the slides. A total of 50 cells were scored in duplicate gels for each sample with Comet assay IV software (Perceptive Instruments, Suffolk, UK) equipped to fluorescence microscope (Leica DMLB, Wetzlar, Germany).

### Micronucleus formation

Micronucleus formation was detected in cells by a rapid flow cytometric method described by Bryce et al.^21^, with slight modifications as described previously^18^. Etoposide (1 µM) was used as positive control and cells in medium, or in medium with blank filter extract, respectively, was negative control. In short, after particle exposure to 10, 50 and 100 µg/mL, the medium was removed, and cells were washed once with ice-cold PBS. Photoactivable Ethidium Monoazide Bromide dye (10 μg/mL, EMA, Invitrogen, Thermo Fisher Scientific, Eugene, OR, USA), prepared in the ice-cold buffer solution (PBS + 2% FBS), was then added to each well (300 μL) followed by incubation on ice for 30 min, 15 cm beneath a cool white light. Cells in each well were washed with 600 μL of cold buffer solution (PBS + 2% FBS). Next, filtered lysis solution I (prepared in Milli-Q water containing: NaCl 0.584 mg/mL; trisodium citrate 1.0 mg/mL; Igepal 0.6 μL/mL; RNase A 100 μg/mL and SYTOX Green 0.5 μM) was added to each well (300 μL) and incubated for 1 h at room temperature. Thereafter, filtered solution II (prepared in Milli-Q water with citric acid 15 mg/mL; sucrose 85.6 mg/mL and SYTOX Green 0.5 μM) was added to each well (300 μL) without discarding solution I, and was allowed to equilibrate at ambient temperature for 30 min. After incubation, the cells were acquired using BD Accuri™ C6 (BD Biosciences, Franklin Lakes, NJ, USA) at 488 nm excitation. EMA-associated fluorescence was recorded in the FL3 channel (610/20 nm) and SYTOX Green fluorescence was collected at FL1 channel (530/30 nm). A total of 10,000 events per sample. Data analysis was performed using the BD Accuri™ C6 Software.

### Occupational exposure measurements

#### The AM facility

The investigated AM facility used PBF-LB with HX and IN939 powder. The printing workshop is approximately 1000 m^2^ divided into sections by 2.5-m-high plastic walls. Three sections are printer areas, with two to four printers in each, one section with the saw, and a section with post-processing, non-destructive testing, ultrasonic cleaning of sieving equipment, packing, and an enclosed CNC machine. The general ventilation had at the time of the present study its air intake on the roof and exhaust at the floor.

The AM process involved manual loading of powder into the printer, the enclosed print process itself, manual taking out the build, depowdering in an enclosed system, removal of base plate by sawing, and eventual grinding. Furthermore, the printer is cleaned after use and unused powder needs to be sieved for re-use. During print, the process is closed and under argon atmosphere.

#### Sampling of total and inhalable dust

Exposure was assessed by gravimetric analysis of total and inhalable dust in parallel since the metals of interest are regulated by Swedish law in these dust fractions. Sampling of total dust was performed according to a modified version of the National Institute of Occupational Safety and Health Manual of Analytical Methods 0500 using an open-faced cassette. Inhalable dust was collected using an IOM sampler (SKC Ltd, Dorset, UK). Both samplers were used with a 25-mm mixed cellulose ester filter and an airflow rate of 2 L/min. For personal exposure measurements, the samplers were placed in the breathing zone, whereas for the stationary measurement, the pumps were placed on the machine work tables, at a height of approximately 1–1.2 m. Filters were analyzed gravimetrically. Metal analysis was performed by means of inductively coupled plasma mass spectrometry (iCAP™ Q; Thermo Fisher Scientific, Waltham, MA, USA).

#### Description of work tasks in focus

Some typical AM work tasks were in focus during the measures. This included a stationary measurement with a sampler right outside the hatch of an AM printer utilizing IN939. The hatch of the machine was opened to take out the build, clean the printer and prepare/start a new print. To compare personal and near-field exposures, personal sampling was performed on the operator performing the tasks specified above. No additional dust-generating work tasks were performed by the operator during the sampling day. The second operator, handling AM machines printing HX, carried pumps and filters. This operator performed four rounds of sieving of HX powder for recycling. This included handling of powder containers and connecting them to sieving equipment followed by cleaning. The remaining time this operator monitored running printing machines but performed no other dust-generating work tasks. The last operator handled the saw that separates ready prints from the base plate. The saw was equipped with local exhaust ventilation in the direction of the blade but were otherwise not enclosed. The operator also performed grinding of prints in local exhaust hoods during the day. All operators wore Powered Air Purifying Respirators fitted with P3 filters during the work task. The efficiency of the masks was investigated by analyzing the content of particles in a used mask using SEM imaging (FEI XL30 SEM and INCA software, 20 kV, Stockholm, Sweden).

#### Particle measurements

Direct-reading particle-counting instruments were used to investigate the operators’ exposure during different work tasks as described above. Two different instruments were used: Lighthouse 3016-IAQ (Lighthouse Worldwide Solutions, CA, USA) measures respirable particles sized between 0.3 and 10 μm, and Aerasense Nanotracer (Philips, Best, the Netherlands) for the detection of ultrafine particles sized between 10 and 300 nm. The particle counters were placed within one meter of the investigated processes.

#### Exposure markers in urine

The workshop had 10 AM operators, whereof 7 (6 females, 1 male, age 25–59 years) volunteered to provide urine samples for exposure assessment. Four of these operators have routine work with printing and sieving, one is a dedicated saw operator and two have various post-processing work tasks, mainly grinding. Controls were obtained from recruited unexposed office workers (n = 7, 4 females, 3 males, age 27–38 years). As described above, all operators wore Powered Air Purifying Respirators fitted with P3 filters during their work tasks. Morning urine was collected in acid-cleaned sampling tubes from AM operators (n = 7) and controls (n = 7) at the beginning and the end of a workweek. Monday samples were frozen until delivery of both Monday and Friday urine. Samples were then kept at − 20 °C until further analysis. Before metal analysis, specific gravity of the urine samples was measured. Samples were diluted ten times and acidified using 0.65% nitric acid (WVR) and metal levels were assessed by ICP-MS (iCAP™ Q; Thermo Fisher Scientific, Stockholm, Sweden). The limits of detection (LOD) were: 0.18 µg Ni/L 0.16 µg Cr/L, 0.001 µg Co/L, 0.2 µg Mo/L, and 2.34 µg Fe/L, and the limits of quantification (LOQ): 0.25 µg Ni/L, 0.18 µg Cr/L, 0.002 µg Co/L, 0.28 µg Mo/L, and 4.02 µg Fe/L.

## Results

### Physical–chemical characterization

**Figure 1:**
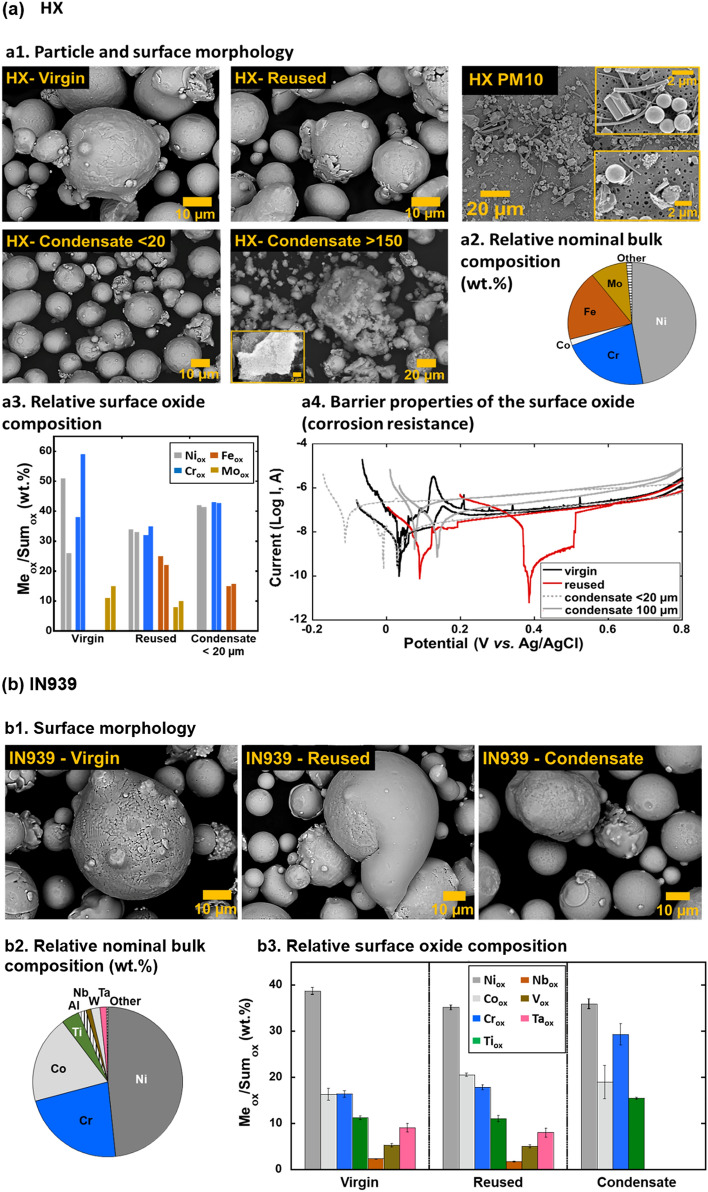
Summary of physical–chemical characteristics (surface morphology, particle size, relative bulk and surface composition), and corrosion characteristics (barrier properties of surface oxide) of Hastelloy X (HX) (**a**) and Inconel 939 (IN939) (**b**) powder particles.

**Figure 2:**
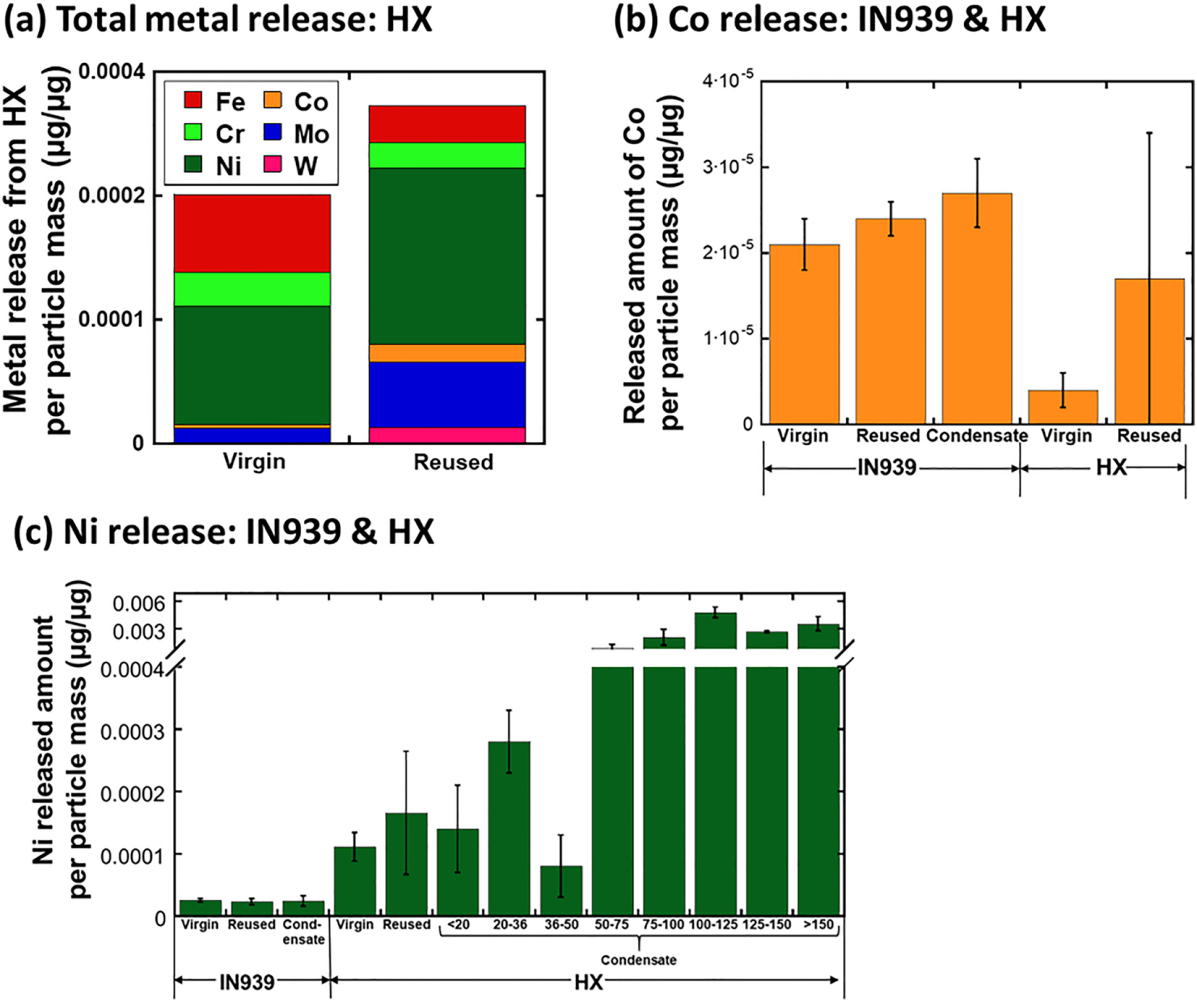
Released amounts of Fe, Cr, Ni, Co, Mo, and W per particle mass (µg/µg) from virgin, reused and condensate Hastelloy X (HX) powders of different sizes (µm) (**a**); released amount of Co (**b**) and Ni (**c**) from the Inconel 939 (IN939) and HX powders after 24 h exposure in ALF (pH 4.5, 37 °C).

Results of the detailed particle and surface characterization of the virgin, reused, condensate, and dust (collected on filter) particles of the nickel-based alloys HX and IN939 are compiled in Fig. 1. Except for particle shape and size, the measurements also involved compositional analysis of the outermost surface as this is the interface towards the environment, i.e., upon cell contact. The extent of dissolved/released metals from the different powders after 24 h in ALF (24 h, 37 °C) is compiled in Fig. 2.

The virgin and reused HX particles showed a relatively spherical morphology with evident dendritic microstructures (Fig. 1a1). Their sizes normally varied between 5 and 50 µm, with adhered smaller sized particles. The reused powder showed smoothened particles with less evident dendritic microstructural features. The condensate particles generally revealed a slightly smaller size compared with the virgin and reused particles. The condensate particle size fraction, > 150 µm (based on the sieving process), contained a lot of comprised “fluffy” nanosized particles attached to larger-sized particles. The dusted HX collected on the filter as PM_10_ showed particles of various shape and sizes of, approximately, less than 10 µm. The collected dust powder also comprised fibers from the filter. According to compositional analysis by means of XPS, all HX particles revealed evident different surface oxide compositions compared to the nominal bulk composition (Fig. 1a2, a3). In addition, a clear composition difference on the outermost surface was observed between virgin and reused powders (Fig. 1a3). The reused HX particles disclosed reduced relative contents of oxidized Cr, Ni and Mo, as well as the presence of oxidized Fe in the outermost surface oxides, which was not observed on the virgin particles. The surface oxide of the condensate HX particles was predominantly composed of oxidized Fe, Ni and Cr, indicating large differences compared to the other HX powders (virgin, reused). According to the XRD measurements on HX condensate powders, no crystalline Cr-rich phase could be observed, only crystalline FeNi (Fig. S1 and Table S1). Cr was for all powders present in its trivalent (III) oxidation state. The barrier properties of the surface oxides from a corrosion perspective in ALF (Fig. 1a4) were generally improved for the reused HX particles (with higher corrosion potential and lower corrosion current) compared with the virgin particles, though with large variations. These findings were in line with the observation of different corrosion properties for the condensate HX powders sieved into different particle size fractions, as exemplified for the < 20 µm and 100–125 µm size fractions. The smaller particles were less corrosion resistant (with lower corrosion potential and higher corrosion current) compared with larger-sized condensate particles, and the virgin and reused powders. Large variations between different measurements reflect the nature of the particles of varying surface composition and particle morphology, as described above. As a consequence, their dissolution pattern varied between the virgin and reused particles and within the sieved condensate particle size-fractions. This is illustrated in Fig. 2a, showing the extent of released metals of the main alloy constituents from the virgin and reused HX powders, in Fig. 2b for the release of Co, and in Fig. 2c for Ni release from the condensate particles of different sieved size fractions. Even though differences could be observed, the extent of particle dissolution was very low (< < 0.06% of the particle mass).

Similar observations were made for IN939 (Fig. 1b) showing different surface oxide composition compared with the bulk composition. However, the reused powder did not show any significant differences in surfaces composition, whereas the composition of the condensate powder was very different with increased relative content of oxidized Cr(III) and Ti and the absence of Nb, V, and Ta oxides observed on the virgin and reused powder particles. Only minor differences in the extent of released Ni and Co were observed between the virgin, reused and condensate IN939 powders (Fig. 2b, c). Compared to the HX alloy, relatively more Co was released from IN939 into the ALF, possibly related to the presence of Co oxides at the outermost surface.

### In vitro cytotoxicity of various HX and IN939 powders

**Figure 3:**
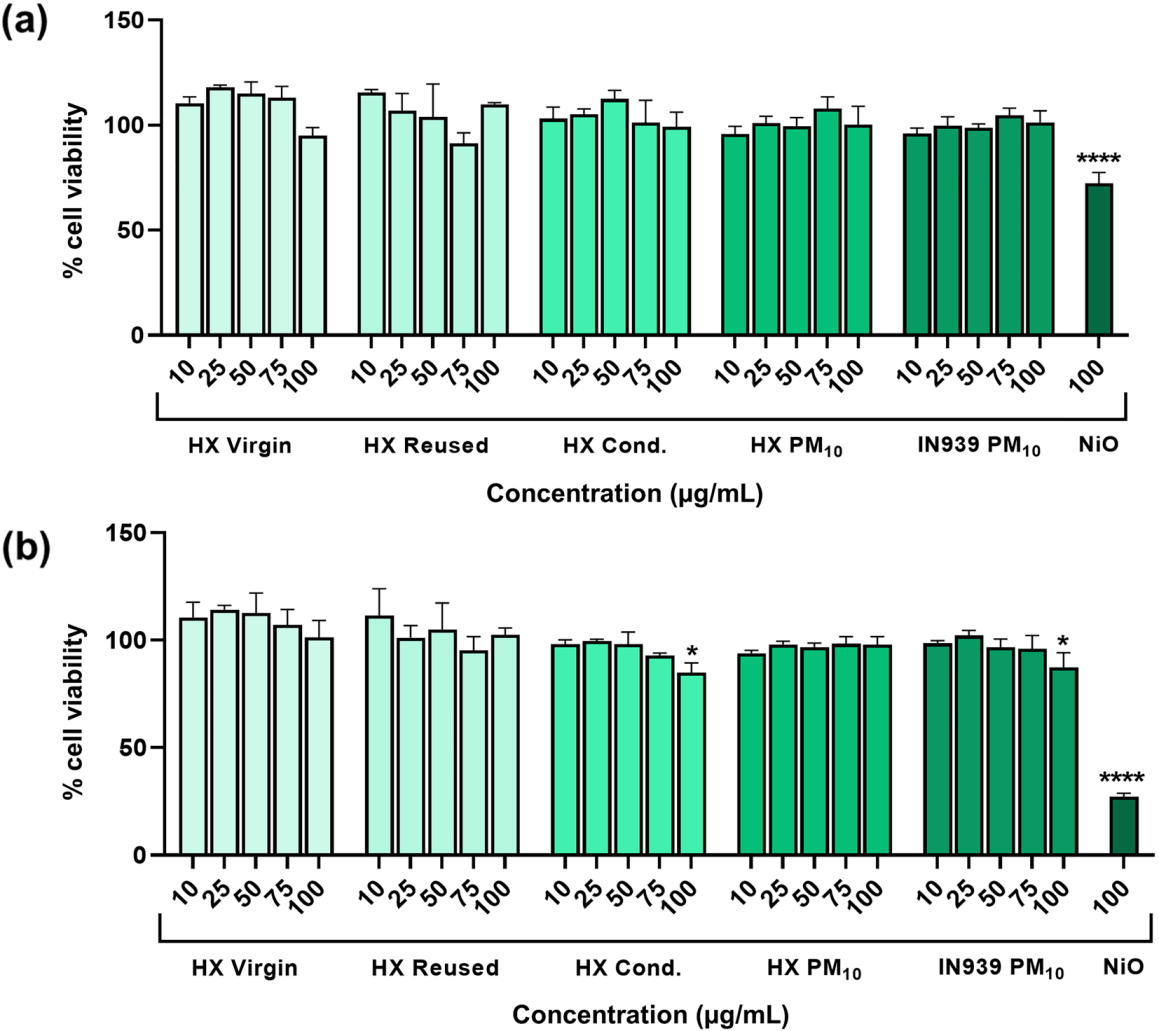
Cell viability assessed by Alamar blue assay following 24 h (**a**) or 48 h (**b**) exposure of HBEC cells to various Hastelloy X (HX) and Inconel 939 (IN939) particles. The bars show % cell viability and negative control cells were set to 100%. Control cells were exposed to cell culture medium for HX virgin, HX Reused and HX Condensate, and to a filter extract (the same volume as the highest dose tested) for the HX and IN939 collected on filter. The results are presented as mean ± SE of three independent experiments (n = 3). Asterisks indicate significance (*P* < 0.05) compared to untreated cells. NiO nanoparticles (100 µg/mL) were used as a positive control.

To investigate cell viability, human lung cells were exposed to five different powder particles: virgin and reused HX, condensate particles formed during HX printing (the condensate sized < 20 µm characterized above) as well as the dusted HX and IN939 particles collected on filters. After HBEC exposure to all tested particles (10–100 µg/mL), none of them were shown cytotoxic, except for a slight reduction in cell viability for the HX condensate and IN939 collected on filter for the highest concentration tested (100 µg/mL) after 48 h exposure (Fig. 3).

### Genotoxicity and oxidative stress of “dusted” HX PM_10_ and HX condensate

**Figure 4:**
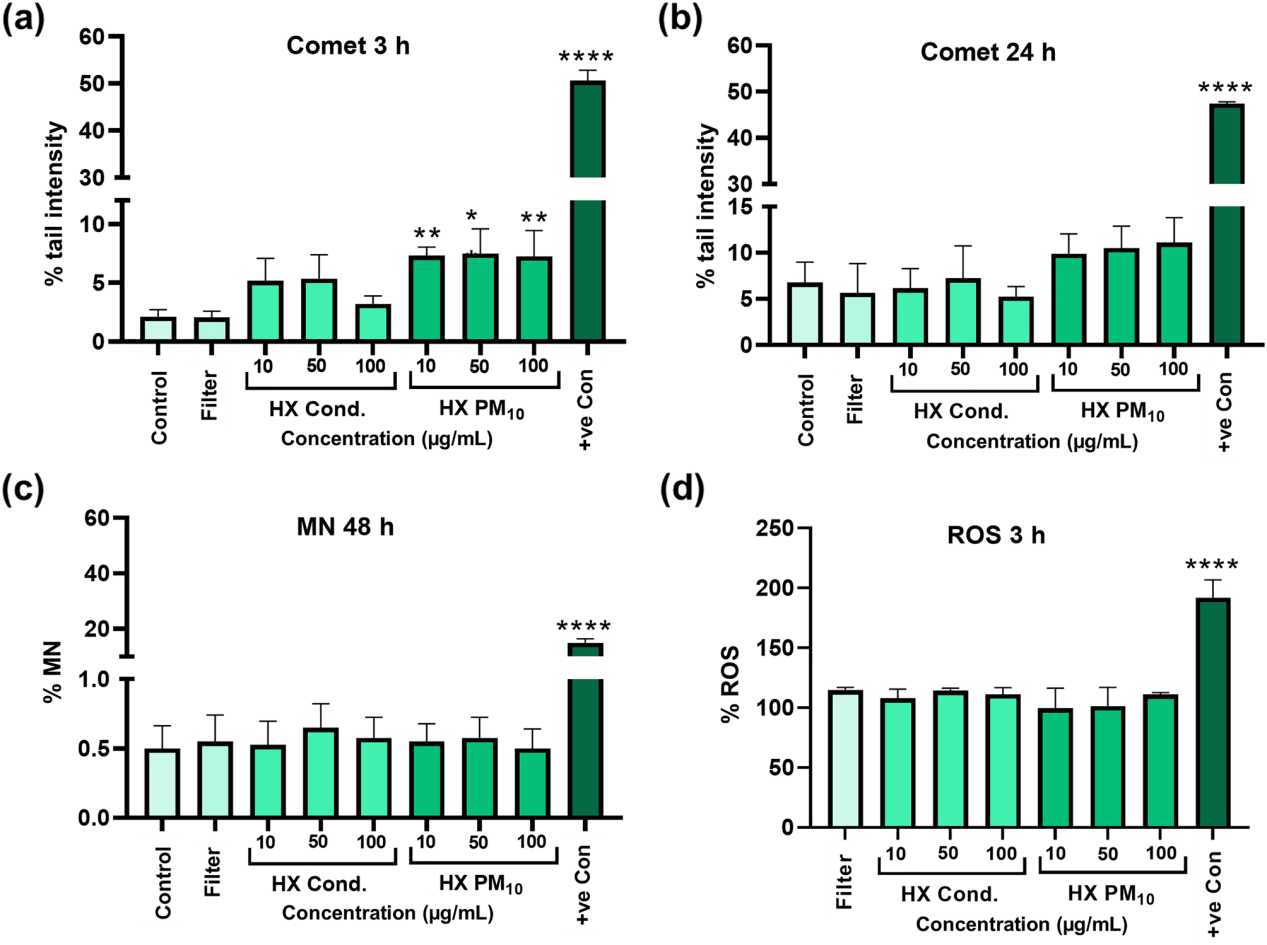
Genotoxicity assessment and intracellular ROS formation after exposure to the Hastelloy X (HX) condensate particles and the dusted HX collected on filters. The comet assay was used following exposure of HBEC cells for 3 h (**a**) and 24 h (**b**). The bars represent the tail intensity (% DNA in the comet tail) and H_2_O_2_ (100 μM for 5 min on ice) was used as a positive control. Micronucleus (MN) formation (%) assessment using flow cytometry following 48 h exposure (**c**). Etoposide (1 µM) was used as positive control. Intracellular ROS formation was determined by using the DCFH-DA assay after 3 h exposure (**d**). Fe_3_O_4_ nanoparticles (50 µg/mL) was used as positive control. The results are presented as mean ± SD of three independent experiments (n = 3). Asterisks indicate significance (*P < 0.05, **P < 0.01) increase compared to untreated cells (negative control, i.e. medium for the condensate and blank filter extract for the dusted HX collected on filter).

Following the initial cytotoxicity testing, the HX condensate and “dusted” HX particles (HX PM_10_) collected on filter were selected for genotoxicity testing by using the comet assay (detecting DNA strand breaks) and the micronucleus assay (detecting chromosomal damage). In addition, the intracellular ROS formation was assessed. The results showed an increase in the DNA strand breaks for the dusted HX collected on filter after 3 h exposure, but there was no dose–response (Fig. 4a). Minor and non-significant increases were observed following exposure to the HX condensate particles at 3 h. The levels were rather similar at 24 h, except for a non-statistically significant increase compared to the filter control, which showed higher variation at 24 h (Fig. 4b). Furthermore, no clear change in micronucleus formation was observed for any of the exposures following 48 h exposure of HBEC cells (Fig. 4c). Finally, in order to investigate whether the small increase in DNA strand breaks noted for HX PM_10_ (Fig. 4a) on filter could be linked to intracellular ROS, the DCFH-DA assay was performed for both the PM_10_ particles and HX condensate particles. The results showed no increased levels of intracellular ROS (Fig. 4d).

### Occupational exposure

Levels of dust, including metal containing particles, were measured in the AM workshop. Stationary measurement levels were in general low (0.17 mg/m^3^ inhalable dust) during printing of IN939 (see Table 1). A low particle exposure during printing was also confirmed by the personal sampling devices. However, the AM operator who was sieving HX had a relatively high exposure of inhalable dust (1.32 mg/m^3^). Metal analysis of this sample showed metal concentrations of 2.9 μg Cr/m^3^, 0.4 μg Co/m^3^, 9.2 μg Ni/m^3^ and 1.5 μg Mo/m^3^ (Table S2). For each metal, this corresponds to less than 2% of the Swedish TWE-OEL (time weighted average-occupational exposure limit) in the inhalable dust fraction.

**Table 1:** Levels of dust and metals in air during different AM tasks.

Sampling location	Total dust* (mg/m^3^)	Inhalable dust (mg/m^3^)	% TWA-OEL Inhalable dust
ST EOS M 290 Printer: printing IN939	< 0.01	0.17	3
PS AM operator: printing IN939	0.09	0.06	1
PS AM operator: sieving HX	0.13	1.32	26
PS Post-process operator: sawing, grinding	0.04	< 0.03	< 1

*TWA-OEL* time weighted average occupational exposure limit (Swedish), *ST* stationary measurement, *PS* personal sampling.

*There is no Swedish TWA-OEL for total dust.

**Figure 5:**
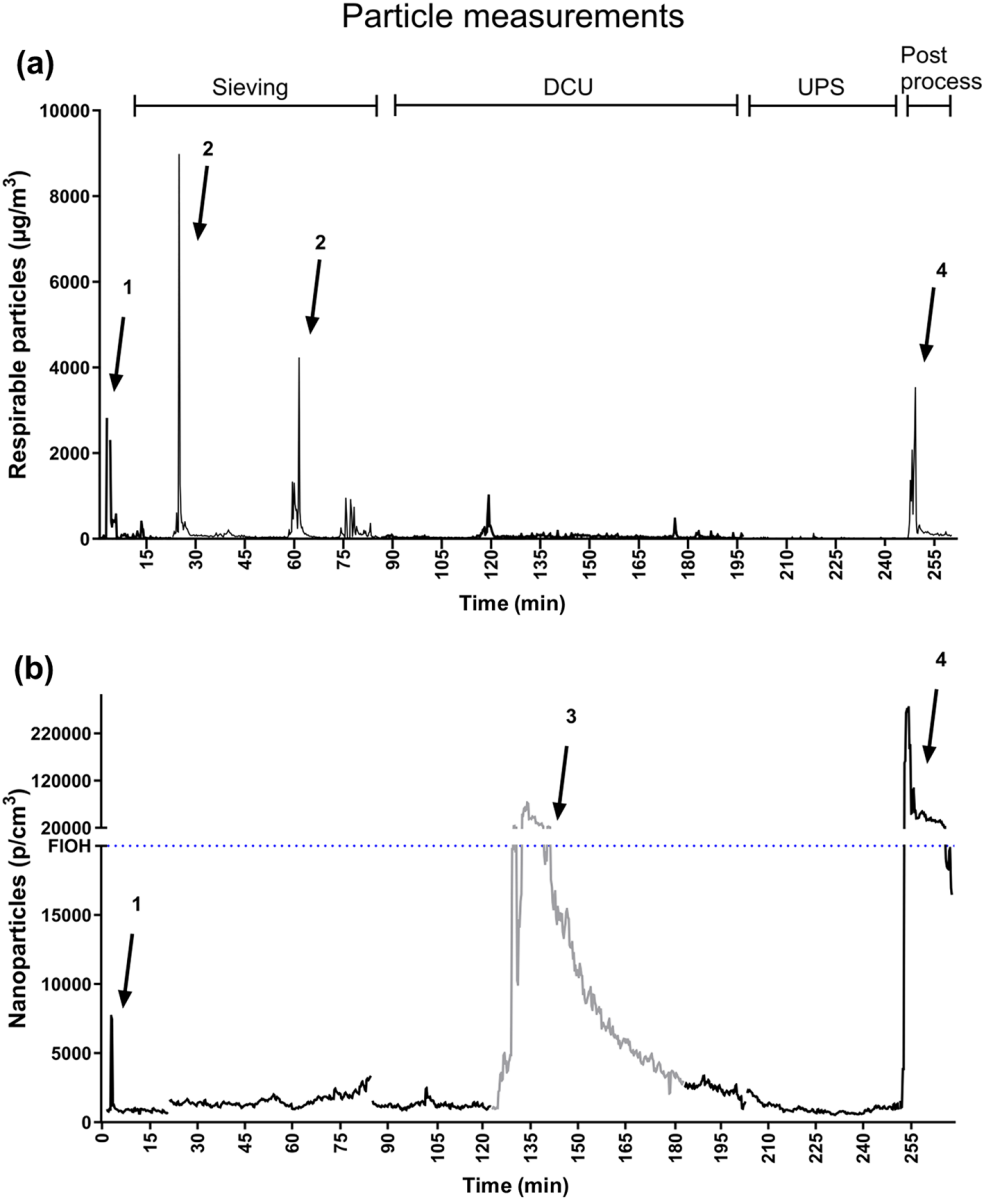
Time-resolved measures of particle concentrations in the AM facility during selected work tasks. The graph shows the total mass of respirable particles in the size range of 0.3–10 µm measured using Lighthouse (**a**) and the number of nanoparticles in the size range of 10–300 nm measured using Aerasense Nanotracer (**b**). The arrows point to: (1) pouring of virgin powder, (2) opening and fastening of the sieving container, (3) particle generation as a result of grinding at a nearby workstation (grey line), and (4) grinding. The dotted line corresponds to the 8 h nanoparticle target levels defined by the Finnish Institute of Occupational Health^34^. DCU: Detail cleaning unit; UPS: Unpacking Station.

Particle counting instruments were used to identify working tasks from which respirable particles (0.3–10 µm, measured with Lighthouse) and ultrafine particles (10–300 nm, measured with Aerasense Nanotracer) were emitted. Based on previous measurements at the same site by the authors^10^ and the following preventive measures carried out by the company, have resulted in enclosed printing processes. Nevertheless, there are still several work tasks in the AM process chain with potential particle exposure, including unpacking the build from non-sintered powder, sieving and processing the powder for re-use, and post processing of the build. The most interesting work tasks from a particle exposure perspective at the investigated company are illustrated in Fig. 5. These tasks include sieving and pouring of powder, two types of enclosed de-powdering company systems (a “Detail Cleaning Unit”, DCU, and an “Unpacking Station”, UPS), and post-processing by sawing and grinding. The background levels of particles were generally low (< 4000 ultrafine particles/cm^3^ and < 0.01 mg/m^3^ respirable particles). However, high transient peaks of respirable particles (> 3 mg/m^3^) were observed when the powder containers were fastened and removed between sieving of batches, as well as when the powder to be sieved was poured openly between the containers. The levels of nanoparticles were low during sieving (< 3500 particles/cm^3^) but increased considerably when the virgin powder was poured at open conditions between the containers prior to sieving (> 12,000 particles/cm^3^). No particle emission was detected from either the two enclosed de-powdering systems. Instead, high levels of nanoparticles, which rapidly spread within the workshop, were observed during grinding at a nearby work station. During post processing including grinding, high levels of both respirable particles (> 3 mg/m^3^) and nanoparticles (> 100,000 particles/cm^3^) were observed. Sawing did not increase the particle levels to any significant extent due to sufficient process ventilation.

Metal levels in urine were determined for a selected number of controls (n = 7) and AM operators (n = 7) as a marker for exposure (Table 2). No significant increase in the metal levels of either Cr, Co, Fe, Mo or Ni in urine was observed when comparing Monday vs. Friday in neither controls or AM operators, nor when comparing controls vs. AM operators. Measured levels were clearly below proposed biomonitoring action limits (Table 2).

**Table 2:** Density adjusted concentrations of metals (Cr, Co, Fe, Mo, Ni) in the urine of AM operators during a week in the AM facility (n = 7) and in the urine of controls (not working in the AM facility) (n = 7).

Metal nmol/L	FIOH ref. unexposed	FIOH action limit	Monday AM operators	Friday AM operators	Monday Controls	Friday Controls
Cr	10	200	11 (1.0–34)	16 (3.0–43)	19 (8.0–52)	25 (16–64)
Co	25	130	5.4 (2.0–26)	4.6 (2.1–20)	4.5 (3.0–7.0)	5.2 (3.0–12)
Fe	–	–	520 (130–1200)	740 (400–1200)	1000 (290–2200)	1400 (980–2800)
Mo	1340	–	500 (200–920)	580 (320–1200)	540 (280–1500)	680 (320–1600)
Ni	50	100	34 (20–53)	35 (22–63)	52 (25–120)	49 (16–93)

Values are geometric mean (minimum–maximum). *FIOH* Finnish Institute of Occupational Health.

### Detection of particles in used masks

**Figure 6:**
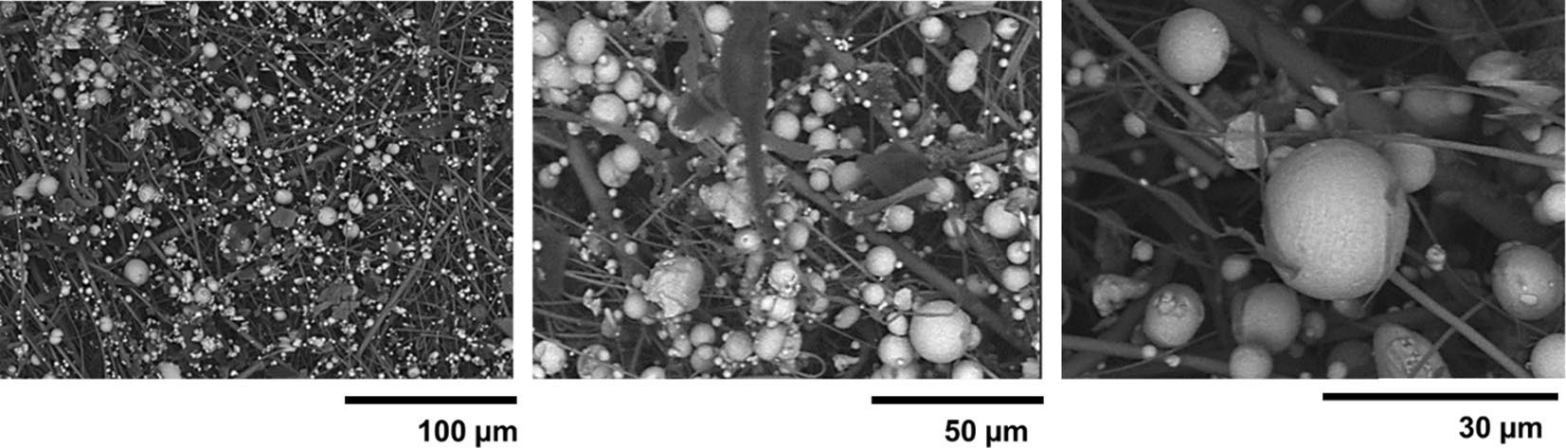
SEM images of metallic airborne particles (containing mainly Cr, Fe, Ni) at an AM site captured by a fresh air filter used by an AM operator for 6 months.

The importance of using powered Air Purifying Respirators to hinder particle inhalation was clearly observed when investigating such a mask used by an AM operator at the site for 6 months. As can be observed in Fig. 6, AM particles of different sizes were clearly captured by the filter.

## Discussion

The aim of this cross-disciplinary study was to increase the understanding of the physical–chemical characteristics, toxicity and occupational exposure of particles used and formed during AM of two nickel-based alloys Hastelloy X and Inconel 939 as a case study. This was studied with a focus on in-depth characterization of virgin and reused powders for PBF-LB printing, as well as of condensates and dusted powder (PM_10_), toxicity testing on cultured lung cells and measurements of occupational airborne exposure levels of particles of different sizes formed during different work tasks in the AM facility (including post-processing).

Except for how processing in general can affect the powder characteristics, e.g. flowability, other important questions are if the laser treatment changes the characteristics of non-fused particles and if the handling of such powders for reuse will influence their toxic properties as well as the final properties of the printed part. In line with previous findings^17^, reused powders of both HX and IN939 showed more smoothened surface morphologies with less evident dendritic microstructural features than the virgin powder, indicative of particle fusion and local melting caused by the laser thermal treatment. The reused powders also had an altered surface oxide composition, being less Cr-rich for the reused- compared to the virgin HX powder, which reduced its electrochemical barrier properties (Fig. 1) and resulted in a slightly higher extent of released metals compared to the virgin powder (Fig. 2). Less evident differences in terms of surface oxide composition and metal release pattern (for Co and Ni) were observed for the virgin and reused IN939 powders. The condensate particles of HX showed very different bulk and surface composition, which resulted in increased levels of released metals (mainly Ni) from HX (in ALF, Fig. 2), whereas the effect was minor for IN939. These results, and previous findings^17^ elucidate that the characteristics of particles used and formed during AM activities are highly dependent on the alloy grade, history of the powder, and the manufacturing process using lasers. The different aspects can result in particles for reuse with altered surface oxide composition, microstructure and surface morphology. These factors govern both the metal release pattern in contact with biological fluids, and the characteristics of printed surfaces. Knowledge on altered powder characteristics as a result of the laser treatment of the PBF-LB process is hence of utmost importance from an AM perspective since it may result in unwanted characteristics of printed parts.

Regarding toxicity and health effects, nickel containing alloys are often seen as problematic due to the high nickel content. Indeed, powders of nickel metal and nickel compounds are according to Regulation (EC) No. 1272/2008 classified as carcinogenic upon inhalation (Cat. 1), able to damage organs upon long exposure, and cause respiratory and skin sensitization (Cat 1)^22,23^. Limited knowledge on health effects is still available for the specific nickel-based alloys focused on in this study. However, useful information may be gained from studies on other alloys such as stainless steel that may contain up to 25% nickel. In a recent review on toxicity and health hazard classification on stainless steel it was in general concluded that this material shows low toxicity^24^. Furthermore, based on various aspects such as the low release of nickel from stainless steels in artificial lysosomal fluid and the lack of (nickel-induced) inflammatory lung effects in an 28-day inhalation study it was also concluded that the data “do not support nickel-related carcinogenicity of stainless steels”^24^.

In a previous study on Inconel 718 (containing 57 wt.% nickel), no release of any significant amounts of nickel or other metals was observed as well as no toxic response^25^. The reason for this was concluded to be due to the high surface passivity of the Inconel powder governed by its chromium-rich surface oxide, making the material behave as oxidized chromium metal. One important question is if this can change depending on size or further activities, such as 3D-printing. An example of how it is stated in a safety data sheet is: “As shipped, these complex alloys in massive form have no known toxicological properties other than causing allergic reactions in individuals sensitive to the metals contained in the alloys. Hazardous fume or dust emissions may be released during remelting, grinding, cutting or welding”^26^. Indeed, welding fume particles, including nickel-containing stainless steel welding fumes, are classified as carcinogenic to humans and show various lung effects^27^, and release of Cr(VI) is believed to be one important mechanism^28^. The toxicological data from the present study showed minor effects in the doses tested for HX condensate and HX PM_10_, although a slight increase in DNA strand breaks was noted. We have previously reported a wide range of effects related to Ni metal and NiO nanoparticles, thus indicating that the size is an important parameter^29,30^. Indeed, Mellin et al.^31^ showed previously the formation of small particles during PBF processing of Inconel 939. The study concluded that small respirable metal particles (approximately 1–2 µm) were generated during processing^31^. Except for Ni, also Cr can induce adverse effects. The toxicity and carcinogenicity of Cr is however primarily caused by the hexavalent form of chromium, Cr(VI), and damage to DNA is a main mode of action^12,32^. The Cr present in the investigated alloys exists either as metallic Cr (in the bulk) or as Cr(III) in the surface oxide as confirmed by means of XPS. Furthermore, the extent of Cr release was limited. A recent study investigating various Cr-containing particles concluded that the cellular damage depends solely on whether or not Cr(VI) is released from the particles^32^. Taken together, the low metal release of the investigated alloys can explain the relatively low toxicity. The strand break formation caused by the “dusted” HX should be noted although no micronuclei formation was observed in line with our previous study^18^.

Our results in this specific work environment showed low exposure to dust and metals during printing and de-powdering. This is connected to several years of systematic efforts in-house regarding preventive measures in the workshop, such as the introduction of enclosed powder handling systems. However, open powder handling still occurs, for example during sieving, which poses an exposure risk. Measured levels of metals in the dust were lower than in our previous study^10^, and believed to be a result of the above-mentioned preventive measures introduced by the company. Our previous study showed that “all personal exposure data complied with their respective Swedish OELs, except one AM operator’s personal inhalable exposure to Co (28.3 µg/m^3^)”^10^. Particle counting instruments showed peak emissions at certain work tasks, particularly during grinding. This clearly highlights the importance of continuous preventive actions and possible task-based personal protection equipment (PPE) where enclosed processes are not yet in place, or not possible. Our results highlighting grinding is in line with findings reported by Jensen et al. in which several work tasks were examined. It was shown that grinding led to the highest increase in particle number concentrations (2.5 × 10^5^ particles/cm^3^)^33^. Metal levels in urine from AM operators in our study were clearly below the biomonitoring action limits proposed by FIOH. This can be compared to our previous results at the same site showing non-significant increase in the metal levels of Co (4.7–7.3 nmol/L), Ni (23.2–33.0 nmol/L), and Cr (1.3–1.8 nmol/L) in the urine of the AM operators at the end of the workweek^10^. It should be noted that the operators wear respiratory protection during all kinds of open powder handling, post-processing and cleaning of equipment.

The investigated AM facility was one of the first in Sweden to develop AM from research level to full-scale serial production. As the number of printing devices increases, so does the risk of airborne particle exposure. Our results suggest generally low emission rates of particles although peaks were observed. The use of masks seems efficient as they clearly captured particles originating from AM (Fig. 6) and low levels of exposure markers in urine were detected. Thus, the risks from AM facilities can clearly be reduced with correct measures.

### Supplementary Information

Supplementary Information.

## Data Availability

The datasets generated during and/or analyzed during the current study are available from the corresponding author on reasonable request.
