# Supplementary material for: Health hazards of particles in additive manufacturing: a cross-disciplinary study on reactivity, toxicity and occupational exposure to two nickel-based alloys
Source: Sci Rep. 2023 Nov 27;13:20846. doi: 10.1038/s41598-023-47884-1 (PMC10682021; doi:10.1038/s41598-023-47884-1)
Supplement: Supplementary file 1 — Supplementary Information. [file 41598_2023_47884_MOESM1_ESM.docx]

**Supporting information**

**Health hazards of particles in additive manufacturing: a cross-disciplinary study on reactivity, toxicity and occupational exposure to two nickel-based alloys**

Hanna L. Karlsson^1^, N.V. Srikanth Vallabani^1^, Xuying Wang^3^, Maria Assenhöj^2^, Stefan Ljunggren^2^, Helen Karlsson^2^ and Inger Odnevall^3,4,5^

*^1^Institute of Environmental Medicine, Karolinska Institutet, SE-171 77 Stockholm, Sweden*

*^2^Occupational and Environmental Medicine Center in Linköping, and Department of Health, Medicine and Caring Sciences, Linköping University, Linköping*

*^3^ KTH Royal Institute of Technology, Division of Surface and Corrosion Science, SE-100 44 Stockholm, Sweden*

*^4^AIMES - Center for the Advancement of Integrated Medical and Engineering Sciences at Karolinska Institute and KTH Royal Institute of Technology, Stockholm, Sweden*

*^5^ Department of Neuroscience, Karolinska Institutet, Stockholm SE-171 77, Sweden*

***Composition of ALF***

ALF is composed of 20.8 g/L citric acid, 6.00 g/L NaOH, 3.21 g/L NaCl, 0.128 g/L CaCl2·H2O, 0.090 g/L disodium tartrate dihydrate, 0.086 g/L sodium pyruvate, 0.085 g/L sodium citrate, 0.077 g/L trisodium citrate dihydrate, 0.071 g/L Na2HPO4, 0.059 g/L glycine, 0.0497 g/L MgCl2, 0.039 g/L Na2SO4, adjusted to pH 4.5 by NaOH.

***X-Ray Diffraction***

**Experimental**: The phase composition and structure of the HX condensate powders were characterized by X-ray Diffraction (XRD) using Bruker D8 ADVANCE diffractometer with a rotating copper anode CuKα radiation (λ=1.5405981 Å). Rietveld refinements were conducted using the HighScore Plus software. The Pseudo-Voigt function was used for peak profile refinement.

**Results**: According to the XRD patterns (Fig. S1), both investigated condensate powder fractions (<20 µm and 100–125 µm) revealed two diffraction peaks assigned to the cubic Fe4 phase, and Fe_x_Ni_y_ phases: tetragonal *P4/mmm* Fe_1_Ni_1_, monoclinic *P1m1* Fe_2.03_Ni_1.97,_ or cubic *Pm-3m* Fe_1_Ni_3_ (Table. S1). Due to peak overlap, the exact phase cannot be determined. No Cr-containing crystalline phases were observed.


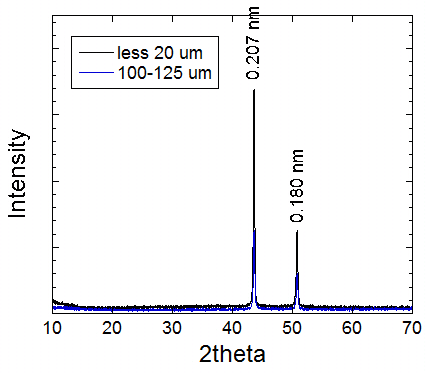


**Figure S1.** XRD patterns of HX condensate powders of two size fractions (<20 µm and 100–125 µm). The d-spacings are given in the figure.

**Table S1.** Characteristic phases corresponding to XRD patterns.

| **Powder** | **Phase** | **Structure** | **Space group** | **Reference** |
| --- | --- | --- | --- | --- |
| less 20 µm,  100–125 µm | Fe4.00 | Cubic | F m -3 m | [1] |
|  | Fe1.00Ni1.00 | Tetragonal | P 4/m m m | [2] |
|  | Ni1.97Fe2.03 | Monoclinic | P 1 m 1 | [3] |

Table S2. Metal levels in inhalable and total dust, µg/m^3^

|  | **Inhalable dust (µg/m^3^)** | | | **Total dust (µg/m^3^)*** | | | |
| --- | --- | --- | --- | --- | --- | --- | --- |
| Metal | TWA-OEL | ST printer EOS M 290  Printing In939 | PS AM operator  Sieving Hx | TWA-OEL | PS AM operator Sieving Hx | PS AM operator Printing In939 | PS Post-process operator sawing, grinding |
| Cr | - | 0.19 | 2.94 | 500 | 0.50 | 0.08 | 0.25 |
| Co | 20 | 0.04 | 0.36 | - | 0.04 | 0.05 | 0.05 |
| Ni | - | 0.16 | 9.17 | 500 | 0.86 | 0.29 | 0.82 |
| Mo | - | 0.01 | 1.53 | 10 000 | 0.10 | <0.01 | 0.13 |
| Mn | 200 | 0.01 | 0.06 | - | 0.03 | 0.02 | 0.01 |

TWA-OEL: Time Weighted Averages occupational exposure limits (Swedish) for elemental metal and inorganic metal compounds; ST: stationary measurement, PS: personal sampling. No metal level was above 2% of its TWA-OEL.

*Modified version of NIOSH method 0500 (PARTICULATES NOT OTHERWISE REGULATED, TOTAL)

**References**

[1] R. Wyckoff, Crystal structures 1 (7–83), American Mineralogist Crystal Structure Database (1963).

[2] R.S. Clarke, E.R. Scott, Tetrataenite—ordered FeNi, a new mineral in meteorites, Am. Mineral. 65(7-8) (1980) 624-630.

[3] T. Tagai, H. Takeda, T. Fukuda, Superstructure of tetrataenite from the Saint Severin meteorite, Zeitschrift fur Kristallographie 210(1) (1995) 14-18.
